# Physiology of peritoneal dialysis; pathophysiology in long-term patients

**DOI:** 10.3389/fphys.2024.1322493

**Published:** 2024-08-13

**Authors:** Raymond T. Krediet

**Affiliations:** Academic Medical Center, Amsterdam, Netherlands

**Keywords:** peritoneal dialysis, pseudohypoxia, glucose, GLUT-1, ultrafiltration failure, free water transportr, peritoneal fibrosis, long-term PD

## Abstract

The microvascular wall of peritoneal tissues is the main barrier in solute and water transport in the initial phase of peritoneal dialysis (PD). Small solute transport is mainly by diffusion through inter-endothelial pores, as is hydrostatic fluid transport with dissolved solutes. Water is also transported through the intra-endothelial water channel aquaporin-1(AQP-1) by a glucose-induced crystalloid osmotic gradient (free water transport). In the current review the physiology of peritoneal transport will be discussed both during the first years of PD and after long-term treatment with emphasis on the peritoneal interstitial tissue and its role in free water transport. Attention will be paid to the role of glucose-induced pseudohypoxia causing both increased expression of fibrogenetic factors and of the glucose transporter GLUT-1. The former leads to peritoneal fibrosis, the latter to a reduced crystalloid osmotic gradient, explaining the decrease in free water transport as a cause of ultrafiltration failure. These phenomena strongly suggest that the extremely high dialysate glucose concentrations are the driving force of both morphologic and functional peritoneal alterations that may develop during long-term PD.

## Introduction

Peritoneal dialysis (PD) as replacement of some aspects of kidney function is limited to removal of potentially toxic solutes that accumulate in severely impaired kidney function, and removal of excess of fluid that can also be present. It should be appreciated that the peritoneum in this condition is not only composed of the mesothelium that covers all abdominal organs, but also includes the submesothelial tissue. This so-called interstitium is composed of a ground substance of hyaluronan and glycosaminoglycans, forming a collagenous network that serves as a skeleton for embedded structures, like microvessels of the circulation and also lymphatic vessels. The interstitium contains a limited number of cells, mainly adipocytes and a few fibroblasts. Overall the interstitium looks rather “empty” at the start of (PD). The mesothelial layer is not a barrier to osmotic fluid transport induced by the glucose content of the intraperitoneal dialysis solution (Flessner, 1994). Therefore, the microvascular wall is likely to be the main barrier for the transport of solutes and fluid from the circulation to the dialysate-filled peritoneal cavity. After a description of the mechanisms of peritoneal transport in the initial phase of PD, the present review will focus on the alterations in peritoneal morphology and transport that can develop in patients after more than 2–4 years of PD, their interrelationships and pathogenesis. Especially the role of the interstitium will be discussed.

## Physiology of solute transport

Solutes can pass the microvascular wall through a system of interendothelial pores. These interendothelial cell clefts comprise 90% of all pores and have radii of about 40 Å (Rippe, 1993). The hydrostatic pressure gradient in the normal situation-so without PD-averages 30 mmHg, but decreases to about zero from the arteriolar to the venous part of the microcirculation (Hall, 2016). Therefore hydrostatic ultrafiltration will be largest in the proximal part of the microcirculation. Using a dialysis solution without an osmotic agent and dextran 70 as intraperitoneal volume marker it could be estimated that peritoneal hydrostatic ultrafiltration in PD patients averages 0.5 mL/min (Struijk et al., 1996). Small solutes like urea, creatinine and glucose have radii of 2 to 3 Å, meaning that their transport through pores of 40 Å is unlikely to be size-selectively hindered and that they pass the small pores by convection to the interstitial tissue. When no PD is applied, the interstitial concentration of these small solutes will be similar to that in the microcirculation, but in case of the presence of a dialysis solution the concentrations of urea and creatinine will be zero. This concentration difference induces diffusion through the same small pore system, which-is a much more efficient process for the removal of small solutes than convection. For instance the solute removal rate averages 17 mL/min for urea and 8 mL/min for creatinine (Smit et al., 2003). As the diffusion rate of a molecule is dependent on its molecular weight, a convection rate of 0.5 mL/min is quantitatively unimportant for small solutes, but this is different for large ones like serum proteins. For instance the transport rate of β_2_-microglobulin from the circulation to the dialysate averages 1 mL/min, half of which is by convection and half by diffusion (Krediet et al., 2022). The diffusion velocity of a solute is dependent on its size, meaning that low molecular weight solutes diffuse faster than those with a higher molecular weight, so urea (MW 60 D)> creatinine (MW 113 D)> glucose (MW 180 D). Besides the concentration gradient between plasma and intra-abdominal dialysate, the small pore density - so the number of perfused peritoneal micro-vessels - is a determinant of over-all diffusion. This parameter is also called the effective peritoneal surface area (EPSA). The dialysate/plasma (D/P) ratio of creatinine is often used as a functional parameter of EPSA. A mean value of 0.72 after a dwell of 4 h has been reported, but with a 95% confidence interval of 0.52–0.90, indicating a marked inter-individual variability (Smit et al., 2003). These values include an intra-individual variability of 7% (Imholz et al., 1998). Based on D/P creatinine, patients have been categorized in four groups: high and low transporters and high and low average transporters (Twardowski et al., 1987). More recently high and low have been changed to fast and slow. The majority of patients are in the average groups, slow transport rates are rare. Morphological studies on relationships between blood vessel density and peritoneal solute transport have shown equivocal results (Sherif et al., 2006; Schaefer et al., 2018; Nakano et al., 2020). An association has been reported in some, but not confirmed in others. A relationship between over-all estimated blood vessel density by sublingual sidestream darkfield imaging and peritoneal solute transport was found for peritoneal glucose absorption in a limited number of PD patients, excluding the fast transporter group (Vlahu et al., 2014), but this needs further confirmation. These findings suggest that in most patients peritoneal transport is dependent on the vascular density, but that fast transporters additionally have peritoneal vasodilation, which will increase EPSA.

## Physiology of fluid transport

Fluid transport during PD is the difference between transcapillary ultrafiltration and back absorption. As stated above, the hydrostatic pressure gradient will induce about 0.5 mL/min ultrafiltration, which is counterbalanced by 0.4 mL/min backfiltration due to the colloid osmotic pressure gradient (4). To withdraw excess fluid from patients with kidney failure additional crystalloid osmosis is applied by the addition of glucose to the dialysis fluid in high doses, leading to an osmolarity of maximal 500 mosmol/L. Part of the glucose will be absorbed during a dialysis dwell. On average this is 60% of the instilled quantity after 4 h. It means that glucose is unable to create a crystalloid osmotic gradient in the small inter-endothelial pores. The presence of the intra-endothelial water channel aquaporin-1 (AQP-1) in peritoneal capillaries and especially venules explains the crystalloid osmotic gradient, because this water channel only allows water to pass and restricts all solutes. So, AQP-1 induces free water transport (FWT) which is different from fluid transport through the small inter-endothelial pores (SPFT) that is composed of water and solutes. The AQP-1 genotype has some effect on its function. The TT genotype is associated with lower ultrafiltration than the CC genotype (Morelle et al., 2021).

FWT will decrease dialysate sodium concentration, the so-called sodium sieving. This is usually assessed after 60 min of a dwell. When the intraperitoneal volume at this time is known, the sodium clearance from the dialysate can be used to calculate FWT and SPFT separately (Smit et al., 2004; La Milia et al., 2005). During the first hour of a hypertonic dialysis dwell 40% of transcapillary ultrafiltration is by FWT and 60% by SPFT (Parikova et al., 2005). The presence of a large EPSA is associated with a high value for SPFT, but with a low FWT and over-all transcapillary ultrafiltration, pointing to the importance of the crystalloid osmotic pressure in peritoneal ultrafiltration (Krediet, 2018).

Due to the problems associated with the extremely high glucose load, glucose polymers have been investigated. Icodextrin is the only available glucose polymer (mainly α1-4 linkages, average molecular weight 16,000 D). The 7% solution is not hypertonic, but induces colloid osmosis, i.e., it induces fluid transport through the small inter-endothelial pores, not through AQP-1. Therefore no sodium sieving is present, and a large EPSA is associated with high ultrafiltration rates (Ho-dac-Pannekeet et al., 1996). The large size of icodextrin molecules imply a slow absorption, making it especially effective during long dwells.

## Early alterations of the peritoneal membrane

Already after a few months peritoneal accumulation of advanced glycosylation end products can be found, first submesothelially, later also perivascular (Yamada et al., 1994). This event is associated with the formation of immature capillaries and with an increase in postcapilillary vessel wall thickness (Nakano et al., 2020). Epithelial-to-mesenchymal transition of mesothelial cells (EMT) is another morphological change. This phenomenon has first been described in 2003 and consists of a transition of the usual epithelial phenotype to a mesenchymal one with loss of cytokeratin expression. EMT in peritoneal biopsies is characterized by the presence of cytokeratin-positive fibroblasts-like cells in the submesothelial interstitial tissue (Yanez-Mo et al., 2003). The prevalence of EMT is highest between 1.5 and 2 years, when it is present in about one-third of patients (Del et al., 2008). Both phenomena may be involved in the early reduction of ultrafiltration, which is the most important functional abnormality during the first years of PD. Indeed a relationship has been found between ultrafiltration and D/P creatinine (Davies, 2004). The relationship between creatinine and ultrafiltration is explained by a similar time course of creatinine appearance in the dialysate and the disappearance of glucose from it, both attributed to diffusion to and from the dialysis solution. The time course of D/P creatinine shows a tendency to increase with time on PD.

## Late alterations of the peritoneal membrane

The early alterations tend to be progressive. In some patients the peritoneum after 4 years is characterized by partial loss of mesothelial cells, submesothelial and interstitial fibrosis, more extensive accumulation of AGEs, and vasculopathy. A schematic overview of these alteration has been published recently (Williams et al., 2002). The blood vessels may show signs of vasculopathy, defined as an increased wall thickness due to subendothelial hyalinosis causing narrowing of the lumen (Selgas et al., 1994). In general, subendothelial hyalinosis of arterioles is mostly seen in patients with diabetes mellitus. In the peritoneum of non-diabetic PD patients subendothelial hyalinosis can be found in all microvessels and is possibly caused by deposition of AGEs sub-endothelialy. This narrowing can progress to partial or total lumen obliteration.

The fibrotic alterations can progress to encapsulating peritoneal sclerosis (EPS) in some patients. Ultrafiltration failure is the most relevant functional abnormality (Selgas et al., 1994; Coester et al., 2014). Impaired ultrafiltration is more severe than expected on the basis of D/P creatinine (Parikova et al., 2022) and concerns both SPFT and FWT (Coester et al., 2014). The role of the expanded interstitium in fluid and free water transport is discussed below. Vasculopathy is likely the cause of the decrease of SPFT, because this pathway is driven by the hydrostatic pressure gradient, that is influenced by the presence of vascular stenosis, similar to the post-stenotic pressure drop in renal artery stenosis.

Patients with EPS have reduced FWT (Sampimon et al., 2011; Morelle et al., 2015). They have normal expression of AQP-1, meaning that the crystalloid osmotic gradient in the fibrotic interstitium surrounding the microvessels must be reduced. Passive diffusion is generally considered the major pathway of the glucose absorption from the dialysis solution during a dwell. But its disappearance from the dialysis solution was faster than expected in an acute model in rats (Park et al., 1999). This suggests additional uptake of glucose in interstitial cells. A glucose molecule is too large to diffuse directly across the cell membrane, but requires the presence of special channels, so-called glucose transporters in the cell membrane. Two types can be distinguished: facilitative glucose transporters (GLUTs) and those that also transport sodium into cells (SGLTs) (Navale and Parajape, 2016). GLUTs facilitate glucose diffusion through the cell membrane, glucose uptake by SGLTs is driven by that of sodium. The presence of both faciliative and sodium dependent glucose transporters in peritoneal tissue has been reported, especially in the mesothelium of patients in the initial phase of PD. Only one study described the presence of glucose transporters in long-term patients in the peritoneal interstitium in the proximity of blood vessels (Schricker et al., 2022). But, expression only gives no information on functionality. Therefore the present review focusses on GLUT-1, of which experimental evidence for function in PD has been shown (Bergling et al., 2022). GLUT-1 is ubiquitously expressed by most cells, among which red blood cells and endothelial cells and also by fibroblasts at a low level, but this is upregulated in the presence of cellular stress, including hypoxia. Increased expression of GLUT-1 by peritoneal interstitial fibroblasts has been postulated as an additional cause of rapid loss of the crystalloid osmotic gradient in long-term patients with extensive peritoneal fibrosis/sclerosis (Krediet, 2021). The functionality of GLUT-1 has been confirmed in a recent study in an acute rat model showing that GLUT-1 inhibition by phloretin improved ultrafiltration (Bergling et al., 2022), while SGLT-2 inhibition had no effect (Martus et al., 2021), thereby supporting the importance of cellular uptake of glucose on the osmotic gradient, irrespective of sodium handling.

## Glucose causes peritoneal damage by pseudohypoxia

**FIGURE 1:**
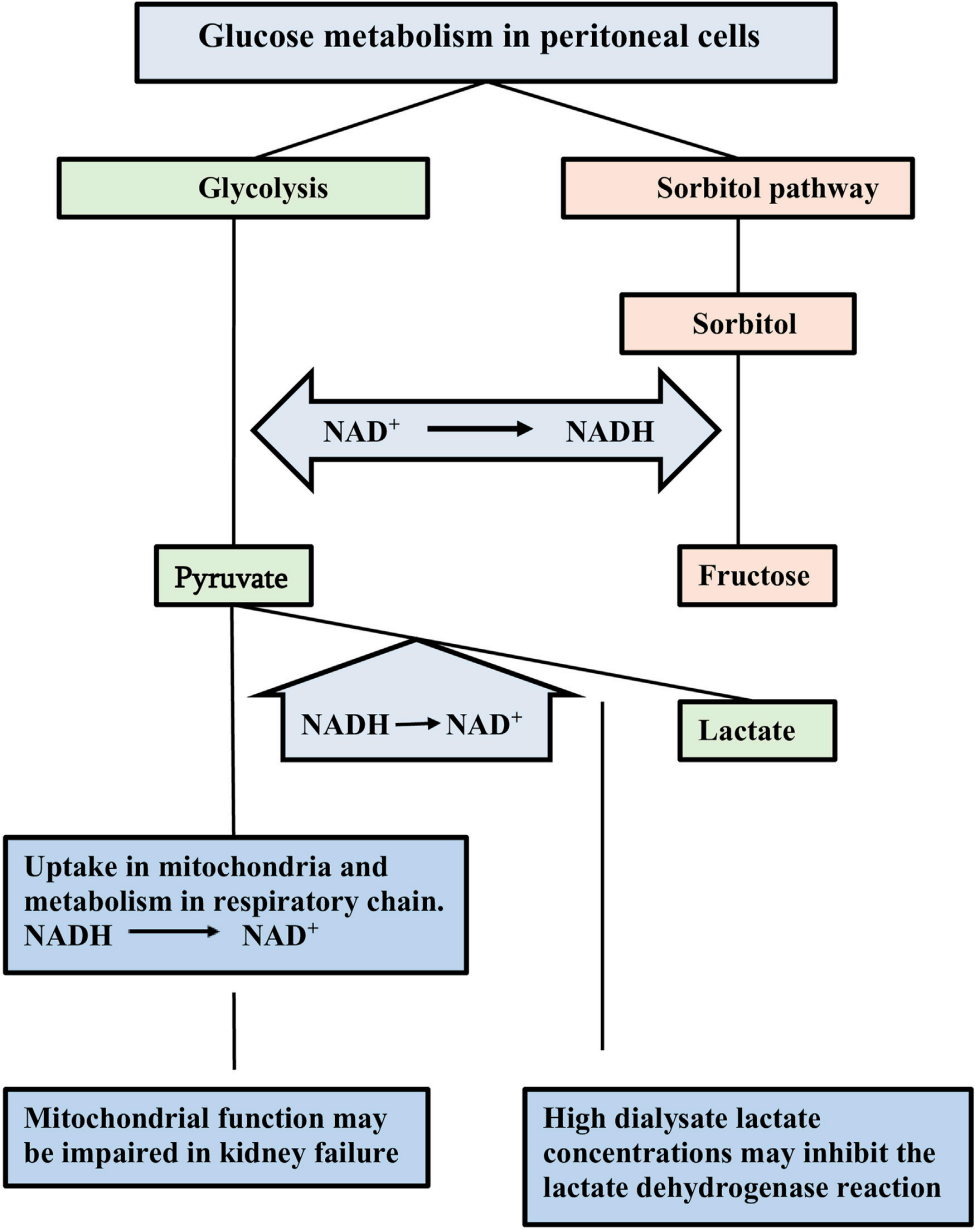
Schematic representation of glucose metabolism in peritoneal cells. Intracellular glucose can be degraded in the glycolysis and in the polyol/sorbitol pathway. Nicototinamide dinucleotide (NAD) is involved in by the reduction of NAD^+^ to NADH. Pyruvate is the end-product in the glycolysis. Oxidation of the formed NADH occurs by metabolism of pyruvate in the mitochondriae and conversion into lactate. These normal compensatory mechanisms may be impaired due to mitochondrial dysfunction and to the use of lactate as buffer in the dialysis solution. Fructose is the end-product of the sorbitol pathway without any compensatory mechanism to oxidize NADH. The resulting increased NADH/NAD^+^ ratio is an indicator of cellular hypoxia. Taken from ref (Krediet, 2022).

Peritoneal micro-inflammation has been considered the cause of the above discussed peritoneal alterations (Lambie et al., 2013). A critical analysis of the available evidence convincingly showed the weakness of this theory (Krediet and Parikova, 2022). Analogously to the genesis of diabetic complications (Williamson et al., 1993), glucose-induced pseudohypoxia is likely to be the driving force of the long-term peritoneal alterations (Krediet and Parikova, 2022). Hypoxia of cells is characterized by an increase in the ratio of reduced/oxidized nicotinamide dinucleotide (NADH/NAD^+^) in the cytosol, due to insufficient supply of oxygen. A similar increase of the NADH/NAD^+^ ratio can occur in situations with a high glucose load, like diabetes mellitus and peritoneal dialysis. This explains the term pseudohypoxia. High extracellular/dialysate glucose concentrations cause glucose transport to the cytosol by glucose transporters. Intracellular glucose is degraded in the glycolysis to pyruvate. NAD^+^ is converted to NADH in this process. The latter can be oxidized to NAD^+^ after uptake of pyruvate in the mitochondria, where it is metabolized in the respiratory chain, and by the lactate dehydrogenase reaction in the cytosol in which pyruvate is converted to lactate. These normal compensatory mechanisms can be impaired in the presence of mitochondrial dysfunction and by using lactate buffered dialysate. In case of a high glucose load additional degradation occurs in the sorbitol pathway, in which glucose is degraded to sorbitol. The latter is broken down to glucose along with the formation of NADH from NAD^+^. The various reactions are discussed in ref (Combet et al., 2000) and illustrated in Figure 1.

## Effects of peritoneal (pseudo)hypoxia on the long-term morphologic alterations

Hypoxia activates the hypoxia inducible factor-1(HIF-1) gene, which causes an upregulation of the genes encoding for various proteins, like vascular endothelial growth factor (VEGF), transforming growth factor β (TGF-β), plasminogen activator inhibitor −1 (PAI-1) and connective tissue growth factor (CTGF). All these have profibrotic and angiogenic properties. Gene expression of VEGF has been shown in peritoneal tissue. It increased with PD duration (Zweers et al., 2001). Such increase was also present for peritoneal effluent VEFF protein (Margetts et al., 2001). TGF-β is probably the most important factor for peritoneal fibrosis (Mateijsen et al., 1999). PAI-1 and CTGF probably exert their effects distal from TGF-β. Besides angiogenic and profibrotic factors, HIF-1 also causes upregulation of GLUT-1, which causes a vicious circle in PD patients: more interstitial GLUT-1 will lead to a progressive decrease of FWT and to more extensive pseudohypoxia and upregulation of HIF-1. This will lead to more fibrosis and angiogenesis. The above mechanism explains the findings that have been reported in PD patients with EPS: the interstitium is composed of dense collagen bundles (Park et al., 1999) and myofibroblasts (Zhang et al., 2023). This hypothesis is supported by the finding that microvessel density and HIF abundance decrease after discontinuation of PD following kidney transplantation (Flessner, 2006).

## Effects of peritoneal (pseudo)hypoxia on peritoneal transport in long-term patients

Angiogenesis, vasculopathy and interstitial fibrosis all with AGE depositions, are the most important morphologic alterations in long-term PD. Ultrafiltration failure is the main functional abnormality. It concerns both free water transport and small pore fluid transport (Sampimon et al., 2011). Small solute transport is somewhat faster than at the start of PD, but the increase can fully be explained by the vascular density. Various theories have been proposed to elucidate the mechanisms of impaired ultrafiltration in interstitial fibrosis, like binding of filtered plasma water by hyaluronan and/or collagen (Wiedner and Wilhelm, 1974). These binding theories are unlikely, because water binding is a process that will get saturated. As stated above, an effect of progressively increased GLUT-1 in interstitial cells is more plausible. The cause of the decrease in small pore fluid transport is not precisely known, but AGE-induced vasculopathy is a possibility, because this will lower the filtration pressure. Interstitial collagen is unlikely to affect peritoneal transport directly, because experiments with a bio-engineerd native collagen membrane offered no indication of hindrance to small solute transport and a linear increase of ultrafiltration with increasing hydrostatic pressure (Toroian et al., 2007). Also another *in vitro* study with a gel filtration column consisting of bovine collagen-1 showed no effects on glucose transport, because it penetrated in the water shield of the fibers (Toroian et al., 2007). These *in vitro* studies support the contention that collagen itself is not important in the long-term alterations in peritoneal transport, but that glucose-induced pseudohypoxia is the driving force.

## Summary and conclusions

The physiology of peritoneal transport in the initial phase of PD is only different from general microvascular filtration of plasma with respect of two conditions: (1) the presence of dialysis fluid in the peritoneal cavity and interstitial tissue allowing diffusion of solutes from the circulation to the dialysate through inter-endothelial pores, and (2) the presence of glucose in high concentrations, that induces free water transport through the intra-endothelial water channel AQP-1, and thereby removal of excess water from the body. Ultrafiltration is determined by the crystalloid osmotic pressure gradient, which decreases during a dialysis dwell due to absorption of the instilled glucose. Impaired ultrafiltration in the initial phase of PD is mainly due to fast transport of small solutes, including a fast glucose absorption rate. Long-term PD affects peritoneal morphology and function. Loss of mesothelial cells, interstitial fibrosis and vasculopathy may develop. Ultrafiltration failure is the most important functional abnormality, not necessarily associated with fast small solute transport rates. It can be caused by an impaired hydrostatic pressure gradient due to vasculopathy, but is often due to high uptake of glucose in interstitial fibroblasts by the glucose transporter GLUT-1. Glucose-induced pseudohypoxia is probably the cause of the increased GLUT-1 expression by HIF-1, that also increases the expression of various other factors involved in angiogenesis and fibrosis. These include VEGF, TGF-β, PAI-1 and CTGF. Glucose exposure in PD increases the cytosolic NADH/NAD^+^ ratio in peritoneal cells. The resulting pseudohypoxia is the driving force for the long-term morphologic and functional alterations.
